# Prevalence of neck/shoulder pain among public hospital workers in China and its associated factors: a cross-sectional study

**DOI:** 10.1038/s41598-020-69382-4

**Published:** 2020-07-23

**Authors:** Hongyun Dong, Qiong Zhang, Guangzeng Liu, Tingguo Shao

**Affiliations:** 1 Shouguang People’s Hospital, NO. 3173 Jiankang Street, Shouguang, Weifang, 262700 Shandong Province China; 2 School of Nursing, Weifang University of Science and Technology, NO. 1299 Jinguang Street, Shouguang, Weifang, 262700 Shandong Province China

**Keywords:** Health care, Health occupations, Risk factors, Signs and symptoms

## Abstract

Studies have reported that neck and/or shoulder pain (NSP) was prevalent and a non-ignorable occupational health problem in healthcare providers. Considering data deficiency on NSP, we aimed to investigate the prevalence and sick leave absence of NSP among public hospital workers in Shandong, China and to explore the associated factors for chronic NSP. A self-administered questionnaire including the Dutch Musculoskeletal Questionnaire and Modified Nordic Musculoskeletal Questionnaire was filled in by 30,520 hospital workers in 37 hospitals selected randomly from among all public hospitals of Shandong, China. The 12-month prevalence of NSP lasting for at least 3 months and sick leave absence due to NSP among 29,547 public hospital workers was 15.6% and 11.4%, respectively, most frequently reported in tertiary hospital workers (27.4% and 18.9%) and clinicians (19.1% and 15.2%). Log-binomial regression analysis revealed that chronic NSP was significantly associated with hospital level, employment position, contract/temporary employment status (vs. permanent), workload (long work hours per week), ergonomic factors (bending the neck forward for long periods of time, twisting the neck for long periods of time) and computer-related factors (prolonged computer-using time daily, the keyboard too close to the edge of the desk).

## Introduction

During the past decades, musculoskeletal disorders (MSDs) have become common and an unfavorable occupational health problem among healthcare providers^1–4^. Studies have shown that MSDs not only could burden society with great costs but also could lead to decreased productivity, illness, disability and other problems^5–8^. A systematic review and meta-analysis^6^ of including 13 cohort studies revealed that subjects with musculoskeletal disorders of neck or back region had a 17% increase on the rate of developing a chronic disease such as cardiovascular disease, cancer and diabetes compared to subjects without (hazard ratio 1.17, 95% confidence interval 1.13–1.22). Neck and/or shoulder pain (NSP), one of MSDs, has also been reported prevalent in healthcare providers and been studied among some occupations in developed and some developing countries. The prevalence of neck pain or discomfort was reported to be 12% among 5269 Taiwanese nurses^2^ to 51.9% among 181 New Zealand nurses^9^ and shoulders pain or discomfort 17% among 5269 Taiwanese nurses^2^ to 48.6% among 347 Iranian hospital nurses^10^. In mainland China, considering the large number of the Chinese population and population aging, more and more medical services are needed. Due to the trust in public hospitals established and policy support for many years, most people would rather go to public hospitals rather than private hospitals when seeking medical service^11^. From January to November in 2019, the number of visits among all the 11,891 public hospitals and 22,081 private hospitals in mainland China was 2889.5 million and 515.3 million, respectively, and the number of discharged patients was 153.1 million and 31.7 million, respectively^11^. But so far there has been limited data on the prevalence, characteristics and associated factors of NSP among Chinese public hospital workers. However, the prevalence of NSP was reported to be very high among some subgroups, 95.1% for neck pain or discomfort among 567 sonographers from 521 medical institutions in 2017^12^, 62.0% and 60.3% for shoulders pain and neck pain among 1017 obstetrics and gynecology practitioners in 2015^13^, and 42.8% and 38.9% for neck pain and shoulders pain among 180 nurses in 2004^14^. In a cross-sectional study conducted in Shandong province, China, in 2018, the prevalence of MSDs of the neck and shoulders region lasting for at least 24 h in the past 12 months was reported to be 52.1% and 47.6%, respectively, among 14,720 healthcare professionals from eight tertiary hospitals^4^. But these studies were either centered on subgroups of hospital workers^12–14^or only included workers in tertiary-level public hospitals^4^.

Many studies^1,15–17^ have revealed that there were a wide range of influencing factors for MSDs including individual (gender, age, smoking), organizational and physical factors (duration of computer work and awkward postures), and workload (work hours). But these factors were mainly for the initial onset of MSDs^15–17^ and it was severe or chronic MSDs, which burden workers’ life and society largely. Researches concerning chronic NSP with a wide range of potential influencing factors such as workload, individual, ergonomic and computer-related factors included and analyzed are quite limited.

In China with the development of economy and demand of authorities, computer has become basic to hospital workers from writing medical records to checking various examination results^18^. In a national survey of Chinese hospital information status^18^, of 1909 hospitals distributed in 31 provincial level administrative regions in mainland China, 1903 (99.69%) have been equipped with computer network systems, which require employees to work with a computer. Computer using occupies physicians a great deal of time. In a cross-sectional study conducted in a US hospital emergency department^19^, the mean percentage of time spent on entering data into computer in a physician’s busy 10-h shift was 43% (95% confidence interval 39–47%) with a pooled weight average time allocations of 44%, significantly much longer than direct patient care (28%) and other activities. Improper computer usage might bring about damage to the neck and shoulders region. Several studies^20–22^ have reported computer work as an associated factor for MSDs. In a study conducted in Beijing, China^23^, the prevalence of work-related MSDs of the neck and shoulder among 720 office workers using a computer as a main working tool was reported to be 55.5% and 50.7%, respectively. In another study^20^ using the Nordic Questionnaire, the 12-month prevalence of neck and shoulder pain was found to be 55% and 38%, respectively, in 1065 subjects working at visual display terminal > 1 h/day. In a study conducted in the UK, eighty-six percent of 175 data processors reported musculoskeletal pain/discomfort, with the highest prevalence rate found for the neck (58%)^21^. But so far to what extent, workload, computer using and ergonomic factors contribute to chronic NSP among Chinese public hospital workers still remains to be illuminated.

In this study, we aimed to explore the prevalence of NSP and sick leave absence due to NSP among public hospital workers and to compare the differences between different hospital-level workers and between different work positions including clinicians, nurses, other healthcare technicians, and managers and support staff. Potential influencing factors including workload, individual, occupational and computer-using factors for chronic NSP among public hospital workers were also explored and evaluated.

## Methods

### Participants

From September to November in 2019, a cross-sectional survey was implemented in Shandong province, China. Based on hospital functions, facilities, technical level and hospital scale, the public hospitals in China were classified into three levels by Health Administration of China, namely, primary hospitals, secondary hospitals and tertiary hospitals^24^. The primary hospital is a grass-roots hospital, or community hospital that directly provides comprehensive services of medical treatment, prevention, rehabilitation and health care for the community, and it is a primary health care institution. The secondary hospital is a regional hospital that provides medical and health services across several communities, and the tertiary hospital is a cross regional, provincial, municipal and nationwide hospital providing medical and health services. First, 26 primary hospitals, 8 secondary hospitals and 3 tertiary hospitals were randomly selected from among its according level hospitals, respectively. Then of the above 37 hospitals selected, the workers in active service and without exclusion criteria were all invited to participate in the survey. Exclusion criteria were: part-time workers, or workers with traffic accident or trauma related to the neck/shoulder region, tumor, congenital diseases related to neck and/or shoulder region such as spinal stenosis, and systemic diseases such as rheumatoid arthritis, diabetes and psychiatric disease. Exclusion criteria were outlined in the informed consent and the workers themselves decided whether to participate in the survey voluntarily. The paper-based questionnaire used in the study was disseminated to every worker at their weekly meeting and collected next day or next meeting. The hospital workers were divided into four groups according to the trial regulations on positions of health care professionals released by China central leading group for the reform of professional titles^25^: clinicians including physicians and surgeons, nurses, other healthcare technicians, and managers and supporting staff. Other healthcare technicians included pharmacist, laboratory personnel, pathologist, sonographer, radiologist, etc. Managers and supporting staff consisted of administrative personal, financial staff, logisticians, etc. In total, 30,520 public hospital workers including 11,593 clinicians, 13,269 nurses, 3443 other healthcare technicians, and 2215 managers and supporting staff, completed our questionnaire and 4839 failed to participate. Of the 30,520 hospital workers, 14,211 workers were from primary-level hospitals, 9998 from secondary hospitals and 6311 from tertiary hospitals. After checking, 973 questionnaires were excluded due to incompletely filling and 29,547 valid questionnaires were analyzed for the study. Thus the response rate of the study was 83.6%.

The study was approved by the Ethics Committee of Shouguang People’s Hospital, Health Commission of Weifang City (wfwsjk_2019_201) and Health Commission of Shandong Province (2016WS0593). All methods in the study were carried out in accordance with the relevant guidelines and regulations of the Declaration of Helsinki and written informed consent was obtained before the hospital workers took part in the survey.

### Questionnaire

A self-administered paper-based questionnaire was developed for the study and was revised after pilot study.

The final questionnaire consisted of three parts. Part one was general information including sex, age, height (cm), weight (kg), educational level (lower than junior college, junior college, bachelor, master or above), smoking (never smoked, ex-smoker, current smoker), and physical exercise in leisure time (never/almost never, sometimes, often).

Part two was NSP information. The information on NSP was assessed by the Nordic Musculoskeletal Questionnaire (NMQ)^26^, which has been systematically translated into the Chinese language and been validated in the Chinese population^27–30^. The participants were asked whether they had experienced pain or discomfort in the neck and/or shoulder region lasting for at least 24 h, 7 days, and 3 months during the previous 12 months. If a participant suffered from pain or discomfort lasting for at least 3 months in the past 12 months in the neck and/or shoulder region, then chronic NSP was considered. Participants suffering from NSP lasting for at least 24 h needed to answer questions on sick leave absence due to NSP (yes or no).

Section three dealt with work characteristics, occupational ergonomic and computer-related factors, mainly derived from the ISO recommendations and the standardized Dutch Musculoskeletal Questionnaire (DMQ)^31^, of which the Chinese version has been validated and widely used in different occupations of Chinese population^30,32^. In this study, the questions which might be associated with NSP were chosen, including questions on force, dynamic and static load, repetitive load, other ergonomic factors, standing, walking, sitting and working in uncomfortable postures. Occupational ergonomic factors were measured using a dichotomous scale (no/yes) and the Chinese version of the DMQ has been detailed in our previous studies^3,4^. Work characteristics included hospital level, work position, and work hours per week (< 40 h, 40–45 h, > 45 h). Computer using information was measured by hospital workers themselves. Distance of the keyboard to the edge of a desk was defined by distance between the g-h point of the keyboard and the edge of the desk.

### Statistical analysis

**Figure 1:**
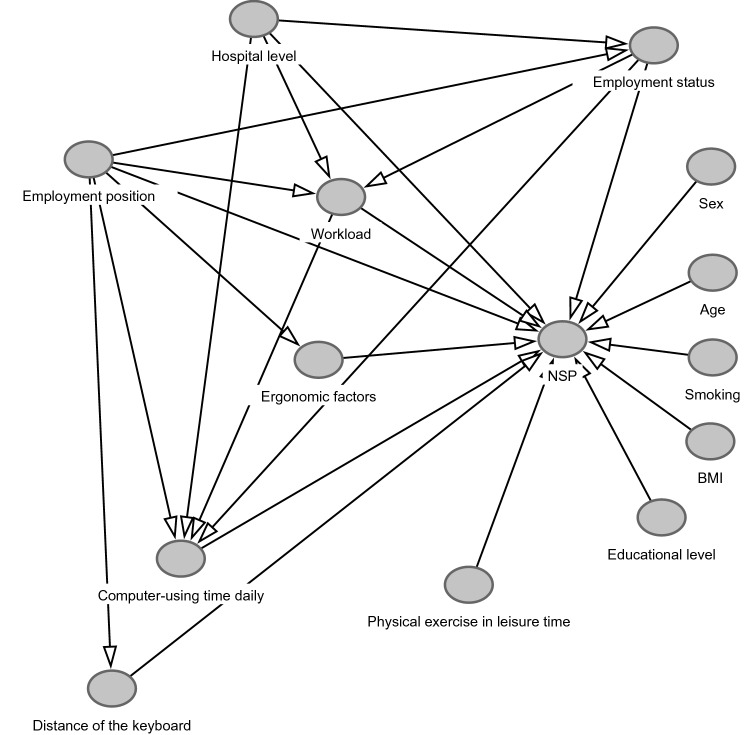
The hypothesized directions of relationship between study variables.

SPSS 25.0 was applied for statistical analysis. Differences between different hospital-level workers and between different groups of hospital workers were analyzed by one-way analysis of variance, chi-square test, or rank sum test. As the odds ratios calculated by logistic regression analysis might overestimate the relative risks when the prevalence was not uncommon, such as over 10%^33–35^, thus log-binomial regression analysis based on generalized linear model was adopted to analyze the association of potential influencing factors including workload, individual, ergonomic and computer-related factors with chronic NSP. Adjusted prevalence ratios (PR) and 95% confidence intervals (95% CI) were calculated in the log-binomial regression analysis with chronic NSP as outcome variable. In order to better identify the confounding variables and estimate the effects of exposure variables, the directed acyclic graph^36–38^ was used. Adjustment for the minimum sufficient set of confounders in the log-binomial analyses was determined by the hypothesized directions of relationship between study variables, depicted in Fig. 1. In model 1 adjusted by age (< 30, 30–40, 40–50, > 50 years old) and sex, hospital level, employment position and other individual factors were considered as the exposure variables and in model 2 adjusted by hospital level, employment position, age and sex, the exposure variables were employment status and other individual factors. In model 3 adjusted by hospital level, employment position, employment status, age and sex, workload, ergonomic factors and other individual factors were the exposure variables and in model 4 adjusted by workload, hospital level, employment position, employment status, age and sex, the exposure variables were computer-related factors and other individual factors. In this study the statistical significance for all tests was set at 0.05.

## Results

The average age of the 29,547 public hospital workers was 37.4 ± 11.2 years old, with 8795 males and 20,752 females. The 12-month prevalence of NSP lasting for at least 24 h, 7 days and 3 months among public hospital workers was 69.7% (69.2–70.2%), 40.7% (40.1–41.3%) and 15.6% (15.2–16.0%), respectively. Of the 29,547 public hospital workers, 3366 (11.4%, 11.0–11.8%) had taken for leave due to NSP in the past 12 months.

The workers in tertiary hospitals were statistically younger than in primary and secondary hospitals and had a statistically higher educational level. The prevalence of NSP lasting for at least 24 h, 7 days and 3 months was 93.9%, 64.8% and 27.4%, respectively, among tertiary hospital workers, 77.4%, 49.1% and 16.9% among secondary hospital workers and 53.0%, 23.4% and 9.3% among primary hospital workers. As to the absence due to NSP, 7.8% (7.3–8.3%) of primary hospital workers had been off duty because of NSP, 11.6% (11.0–12.2%) of secondary hospital workers, and 18.9% (17.9–19.9%) of tertiary hospital workers. For more details, see Table 1.

**Table 1:** General information and NSP characteristics of public hospital workers.

	Total	In primary hospitals	In secondary hospitals	In tertiary hospitals
Questionnaires collected	30,520	14,211	9998	6311
Failed to participate	4839	2198	1603	1038
Valid questionnaires	29,547	13,537	9759	6251
Age	37.4 ± 11.2	38.4 ± 10.1	37.1 ± 11.5	35.7 ± 12.7
Sex				
Male	8795 (29.8%)	3953 (29.2%)	2905 (29.8%)	1937 (31.0%)
Female	20,752 (70.2%)	9584 (70.8%)	6854 (70.2%)	4314 (69.0%)
Marital status				
Never married	4391 (14.9%)	1972 (14.6%)	1446 (14.8%)	973 (15.6%)
Married/cohabiting	23,809 (80.6%)	10,962 (81.0%)	7869 (80.6%)	4978 (79.6%)
Divorced/separated/widowed	1347 (4.6%)	603 (4.5%)	444 (4.5%)	300 (4.8%)
BMI				
< 18.5 (Underweight)	1537 (5.2%)	731 (5.4%)	506 (5.2%)	300 (4.8%)
18.5–23.9 (Normal weight)	18,607 (63.0%)	8551 (63.2%)	6140 (62.9%)	3916 (62.6%)
24.0–27.9 (Overweight)	5525 (18.7%)	2506 (18.5%)	1839 (18.8%)	1180 (18.9%)
≥ 28.0 (Obesity)	3878 (13.1%)	1749 (12.9%)	1274 (13.1%)	855 (13.7%)
Educational level				
Lower than junior college	16,760 (56.7%)	8142 (60.1%)	5563 (57.0%)	3055 (48.9%)
Junior college	4906 (16.6%)	2351 (17.4%)	1620 (16.6%)	935 (15.0%)
Bachelor	4762 (16.1%)	1972 (14.6%)	1580 (16.2%)	1210 (19.4%)
Master or above	3119 (10.6%)	1072 (7.9%)	996 (10.2%)	1051 (16.8%)
Smoking				
Never smoked	20,730 (70.2%)	9415 (69.6%)	6862 (70.3%)	4453 (71.2%)
Ex-smoker	2781 (9.4%)	1251 (9.2%)	917 (9.4%)	613 (9.8%)
Current smoker	6036 (20.4%)	2871 (21.2%)	1980 (20.3%)	1185 (19.0%)
Exercise in leisure time				
Never/almost never	7091 (24.0%)	3172 (23.4%)	2374 (24.3%)	1545 (24.7%)
Sometimes	14,108 (47.7%)	6512 (48.1%)	4640 (47.5%)	2956 (47.3%)
Often	8348 (28.3%)	3853 (28.5%)	2745 (28.1%)	1750 (28.0%)
Work hours per week				
< 40	9050 (30.6%)	4567 (33.7%)	2855 (29.3%)	1628 (26.0%)
40–45	11,814 (40.0%)	5231 (38.6%)	3927 (40.2%)	2656 (42.5%)
> 45	8683 (29.4%)	3739 (27.6%)	2977 (30.5%)	1967 (31.5%)
NSP lasting for at least				
24 h	20,601 (69.7%, 69.2–70.2%)	7179 (53.0%, 52.2–53.8%)	7551 (77.4%, 76.6–78.2%)	5871 (93.9%, 93.3–94.5%)
7 days	12,016 (40.7%, 40.1–41.3%)	3169 (23.4%, 22.7–24.1%)	4795 (49.1%, 48.1–50.1%)	4052 (64.8%, 63.6–66.0%)
3 months	4624 (15.6%, 15.2–16.0%)	1258 (9.3%, 8.8–9.8%)	1652 (16.9%, 16.2–17.6%)	1714 (27.4%, 26.3–28.5%)
Absence due to NSP	3366 (11.4%, 11.0–11.8%)	1054 (7.8%, 7.3–8.3%)	1129 (11.6%, 11.0–12.2%)	1183 (18.9%, 17.9–19.9%)

The average age of 11,251 clinicians was 36.3 ± 11.2 years old including 4902 males and 6349 females. The prevalence of NSP lasting for at least 3 months were 19.1% (18.3–19.8%) among clinicians, 15.0% (14.4–15.6%) among nurses, 11.0% (10.0–12.1%) among other healthcare technicians, and 8.7% (7.5–9.9%) among managers and supporting staff. The prevalence of sick leave absence due to NSP in the preceding 12 months was 15.2% (14.5–15.8%) among clinicians, 10.5% (9.9–11.0%) among nurses, 6.7% (5.9–7.6%) among other healthcare technicians, and 4.5% (3.6–5.4%) among managers and supporting staff. For more details, see Table 2.

**Table 2:** General information and NSP characteristics between different work positions.

	Clinicians	Nurses	Other healthcare technicians	Managers and supporting staff
Questionnaires collected	11,593	13,269	3443	2215
Failed to participate	1868	2085	539	347
Valid questionnaires	11,251	12,794	3349	2153
Age	36.3 ± 11.2	38.1 ± 9.5	37.2 ± 13.8	39.2 ± 15.1
Sex				
Male	4902 (43.6%)	821 (6.4%)	1896 (56.6%)	1176 (54.6%)
Female	6349 (56.4%)	11,973 (93.6%)	1453 (43.4%)	977 (45.4%)
BMI				
< 18.5 (Underweight)	250 (2.2%)	932 (7.3%)	181 (5.4%)	174 (8.1%)
18.5–23.9 (Normal weight)	7818 (69.5%)	7273 (56.8%)	2115 (63.2%)	1401 (65.1%)
24.0–27.9 (Overweight)	1875 (16.7%)	2605 (20.4%)	620 (18.5%)	425 (19.7%)
≥ 28.0 (Obesity)	1308 (11.6%)	1984 (15.5%)	433 (12.9%)	153 (7.1%)
Educational level				
Lower than junior college	4382 (38.9%)	9836 (76.9%)	1404 (41.9%)	1138 (52.9%)
Junior college	2668 (23.7%)	1033 (8.1%)	794 (23.7%)	411 (19.1%)
Bachelor	2613 (23.2%)	1021 (8.0%)	728 (21.7%)	400 (18.6%)
Master or above	1588 (14.1%)	904 (7.1%)	423 (12.6%)	204 (9.5%)
Smoking				
Never smoked	6794 (60.4%)	10,965 (85.7%)	1643 (49.1%)	1328 (61.7%)
Ex-smoker	1149 (10.2%)	787 (6.2%)	442 (13.2%)	403 (18.7%)
Current smoker	3308 (29.4%)	1042 (8.1%)	1264 (37.7%)	422 (19.6%)
Exercise in leisure time				
Never/almost never	1874 (16.7%)	3851 (30.1%)	871 (26.0%)	495 (23.0%)
Sometimes	5864 (52.1%)	5643 (44.1%)	1557 (46.5%)	1044 (48.5%)
Often	3513 (31.2%)	3300 (25.8%)	921 (27.5%)	614 (28.5%)
Work hours per week				
< 40	2873 (25.5%)	3729 (29.1%)	1323 (39.5%)	1125 (52.3%)
40–45	4405 (39.2%)	4937 (38.6%)	1586 (47.4%)	886 (41.2%)
> 45	3973 (35.3%)	4128 (32.3%)	440 (13.1%)	142 (6.6%)
Computer using time daily				
< 3 h	3042 (27.0%)	8355 (65.3%)	1145 (34.2%)	851 (39.5%)
3–6 h	4638 (41.2%)	2984 (23.3%)	1226 (36.6%)	754 (35.0%)
> 6 h	3571 (31.7%)	1455 (11.4%)	978 (29.2%)	548 (25.5%)
NSP lasting for at least				
24 h	8797 (78.2%, 77.4–79.0%)	8899 (69.6%, 68.8–70.4%)	1864 (55.7%, 54.0–57.3%)	1041 (48.4%, 46.2–50.5%)
7 days	5307 (47.2%, 46.2–48.1%)	5135 (40.1%, 39.3–41.0%)	1110 (33.1%, 31.5–34.7%)	464 (21.6%, 19.8–23.3%)
3 months	2144 (19.1%, 18.3–19.8%)	1923 (15.0%, 14.4–15.6%)	369 (11.0%, 10.0–12.1%)	188 (8.7%, 7.5–9.9%)
Absence due to NSP	1705 (15.2%, 14.5–15.8%)	1338 (10.5%, 9.9–11.0%)	226 (6.7%, 5.9–7.6%)	97 (4.5%, 3.6–5.4%)

In model 1, log-binomial regression analysis revealed that higher prevalence of chronic NSP was found in secondary hospital workers and tertiary hospital workers, compared with primary hospital workers, and the prevalence of chronic NSP was also associated with employment position (nurses vs. clinicians, other healthcare technicians vs. clinicians, managers and supporting staff vs. clinicians). In model 2, we found contract/temporary hospital workers had higher prevalence of chronic NSP than permanent ones. In model 3, chronic NSP among public hospital workers was found significantly associated with long work hours per week (40–45 vs. < 40, > 45 vs. < 40), bending the neck forward for long periods of time and twisting the neck for long periods of time. In model 4, the keyboard too close to edge of the desk (< 15 cm vs. ≥ 15 cm) and prolonged computer-using time daily (> 6 h vs. < 3 h, 3–6 h vs. < 3 h) was found to be associated with the prevalence of chronic NSP. For more details, see Table 3.

**Table 3:** Log-binomial regression analysis of chronic NSP associated factors among public hospital workers.

Factors	Chronic NSP n (%)	PR	95% C.I
**Model 1**			
Hospital level			
Primary hospital	1258 (9.3%)	1	
Secondary hospital	1652 (16.9%)	1.9	1.2–2.9
Tertiary hospital	1714 (27.4%)	3.0	1.8–5.0
Employment position			
Clinicians	2144 (19.1%)	1	
Nurses	1923 (15.0%)	0.7	0.6–0.9
Other healthcare technicians	369 (11.0%)	0.5	0.3–0.8
Managers and supporting staff	188 (8.7%)	0.4	0.2–0.7
**Model 2**			
Employment status			
Permanent	2194 (12.4%)	1	
Contract/temporary	2430 (20.4%)	1.6	1.1–2.4
**Model 3**			
Workload			
Work hours per week			
< 40	813 (9.0%)	1	
40–45	1873 (15.9%)	1.7	1.1–2.4
> 45	1938 (22.3%)	2.3	1.4–3.7
Ergonomic factors			
Bending the neck forward for long periods of time			
No	3054 (12.4%)	1	
Yes	1570 (32.0%)	2.4	1.4–4.0
Twisting the neck for long periods of time			
No	3472 (13.2%)	1	
Yes	1152 (36.0%)	2.6	1.6–4.1
**Model 4**			
Computer-related factors			
Distance of the keyboard			
0 = ≥ 15 cm	2104 (11.8%)	1	
1 = < 15 cm	2520 (21.4%)	1.8	1.2–2.7
Computer-using time daily			
< 3 h	1173 (8.8%)	1	
3–6 h	1640 (17.1%)	1.9	1.1–3.2
> 6 h	1811 (27.6%)	3.0	1.9–4.8

Model 1 was adjusted by age (< 30, 30–40, 40–50, > 50 years old) and sex. Model 2 was adjusted by hospital level, employment position, age and sex. Model 3 was adjusted by hospital level, employment position, employment status, age and sex. Model 4 was adjusted by workload, hospital level, employment position, employment status, age and sex.

## Discussion

In this study we found that the prevalence of various types of NSP, including chronic NSP and sick leave absence due to NSP, among public hospital workers was quite common, most frequently reported in tertiary hospital workers and clinicians. The following factors were found to be associated with chronic NSP among public hospitals workers: workload (long work hours per week), ergonomic factors (bending the neck forward and twisting the neck for long periods of time) and computer-related factors (prolonged computer-using time daily and the keyboard too close to the edge of a desk), which provided some further evidence for the influencing factors for chronic NSP among hospital workers.

Different from previous studies^12–14^ mainly centered on subgroups of hospital workers, this is the first large-scale study concerning NSP prevalence, sick leave absence and associated factors among public hospital workers in China with different hospital-level workers and a wide range of possible influencing factors included. The workers were randomly sampled from among all public hospitals in Shandong, China, by stratified cluster sampling method and the response rate of the study was 83.6%, which improves the generalizability of the results. As the study participants were limited to public hospital workers only, thus whether the results apply to all hospital workers, especially private hospital workers, remains problematic. Limitations of the study also included the self-report outcomes and recall information. Although the questionnaire used in the study was from validated and contextualized Chinese version questionnaires^27–30,32^, the direction of the bias in the study could go in either direction if the associated factors were differentially misclassified by those with and without NSP. The cross-sectional design of the study made it difficult to conclude a strong causal relationship between chronic NSP and the associated factors screened out. For example, bending the neck forward and twisting the neck for long periods of time might be a consequence or adaptation to chronic NSP, as the cross-sectional design does not allow to distinguish the temporality between exposure and outcome. Likewise, persons with chronic NSP may be more likely to be employed on a temporary contract. A prospective cohort study might make up the shortcomings and be needed in the future. The wide exclusion criteria might have the study underestimate the NSP prevalence due to the fact that any of the exclusion criteria, such as diabetes and psychiatric disease, might be caused by being a public hospital worker and could lead to NSP. Besides, possible influencing factors occurring outside the work environment, such as smart phone use and sleep habits, and psychosocial factors which might be associated with chronic NSP, were not collected, which might bias our results in the form of residual confounding.

In our study, we found the prevalence of NSP was quite common, especially statistically higher among tertiary hospital workers than primary hospital workers and secondary hospital workers, and higher among clinicians than nurses, other healthcare technicians and managers and supporting staff. In systematic reviews and meta-analyses, the 1-year prevalence of neck pain was reported to be 38–73% among athletes^39^, 45% among midwives, nurses and physicians^40^, 42–83% among hospital physicians^41^, 27.1–47.8% among nurses^42^ and 60% (47–72%) among 1921 physicians^43^, and the prevalence of shoulders pain was 52% (43–61%) among 1360 physicians^43^, similar with or lower than our results. Although many factors including measurement tools were different, the prevalence found in our study was quite high and it is of great importance to pinpoint the risk factors for NSP among public hospital workers, especially tertiary hospital workers and clinicians. As in previous studies the risk factors were mainly for the initial onset of MSDs and acute or short-term MSDs have a tendency to be self-limiting^44,45^, e.g., about 50% of low back pain would recover within 2 weeks and 90% within 6 weeks^46–50^, the evidence about risk factors for chronic MSDs was scarce. Thus, we analyzed the associate factors of chronic NSP other than the initial onset of NSP in this study.

In this study we found many factors, including workload, individual, ergonomic and computer-related factors, were associated with chronic NSP. Firstly, our study found prolonged computer-using time daily was associated with chronic NSP with an adjusted PR of 2.7 (1.7–4.3) for > 6 h vs. < 3 h. Consistent with our study, another cross-sectional study in Germany also found that the duration of visual display terminal work had a significant effect on the neck disorders in subjects performing such work > 6 h per day^20^. A large quantity of work among clinicians was done based on computers such as writing medical records, making diagnoses, prescribing medicine, giving treatments other than based on papers. The computer-using time of clinicians was statistically longer than nurses and other healthcare providers (Table 2), which may to some extent explain the high prevalence of chronic NSP among clinicians. Secondly, our study found that poor placement of the keyboard was associated with chronic NSP. Workers with a smaller distance between the keyboard and the edge of the desk tended to suffer form chronic NSP, indicating that less or no support for the forearms might contribute to chronic NSP. Thirdly, bending the neck forward and twisting the neck for long periods of time were associated with chronic NSP, similar with the results of a systematic review and meta-analysis^51^ which reported that adults with neck pain was correlated with increased forward head posture when compared to asymptomatic adults. At same time we also need to note that bending the neck forward and twisting the neck for long periods of time might be associated with pain measures^51^ and need to be identified for chronic NSP in the future in cohort studies although correlation between them was found in the study. Fourthly, our study showed that hospital workers with temporary/contract employment status were more prone to suffering from chronic NSP than those with permanent employment status. In an Italian longitudinal study^52^, a negative association between health and temporary employment status was found and temporary employment was found to be particularly damaging for a person’s health when prolonged over time. Hospital workers with temporary/contract employment status might be inclined to work harder and longer due to various unmeasured reasons such as low job stability and high financial stress, which might lead to the development or aggravation of chronic NSP. Therefore, intervention measures might be suggested in temporary/contract workers of large quantity in public hospitals.

## Conclusions

The NSP prevalence was quite high among Chinese public hospital workers, especially among tertiary hospital workers and among clinicians. A variety of factors, including workload, ergonomic, individual and computer-related factors, were associated with chronic NSP among public hospital workers. Attention might be paid to the above factors screened out when exploring preventive measures for NSP.

## Data Availability

Data will not be shared publicly but could be accessed upon reasonable request from the corresponding author (ayouwang@163.com).
